# Response to “caution regarding interpretations of intrauterine γδ T cells in protection against experimental vaginal candidiasis”

**DOI:** 10.1038/s41385-021-00396-5

**Published:** 2021-03-17

**Authors:** L. Monin, A. Hayday

**Affiliations:** 1 ImmunoSurveillance Lab, The Francis Crick Institute, London, UK; 2 Peter Gorer Department of Immunobiology, School of Immunology and Microbial Sciences, King’s College London, London, UK

We welcome Dr. Fidel’s interest in our study and the opportunity that this open forum provides for appropriate scientific discourse.

γδ T cells are evidently enriched at barrier sites, where they have been assigned roles in immunoprotection, immunoregulation, and tissue repair. Nonetheless, most studies describing this have focussed on skin and gut, leaving uncertain the cells’ status in the female reproductive tract (FRT), a site where infection, transformation, and tissue remodelling are common. This was the subject addressed by our recent paper in *Mucosal Immunology*.^1^ Most often, science moves forward in small steps. Nonetheless, the data in our paper was sufficient to justify five key conclusions. First, in contrast to an initial report,^2^ and to the general perception that report has underpinned^3^ murine uterine γδ T cells constitute a stromal, not an intraepithelial population of T cells. Second, most murine uterine T cells predominantly express an invariant Vγ6Vδ1 TCR rearrangement and although this is shared with γδ T cells at other sites, e.g. the lung^3^ the uterine cells display distinct phenotypic traits and effector potentials. Third, the murine uterine cells populate the uterus early in life and decrease in number as mice age. Fourth, and in contrast to some other tissue-associated Vγ6Vδ1^+^ cells,^4^ murine uterine γδ T cells develop in a microbiome-independent manner; and fifth, although dispensable for the establishment of pregnancy, γδ T cells can play a non-redundant role in the protection of the FRT from vulvovaginal candidiasis (VVC). It is with respect to this last conclusion that Dr. Fidel has raised several issues.

**Fig. 1:**
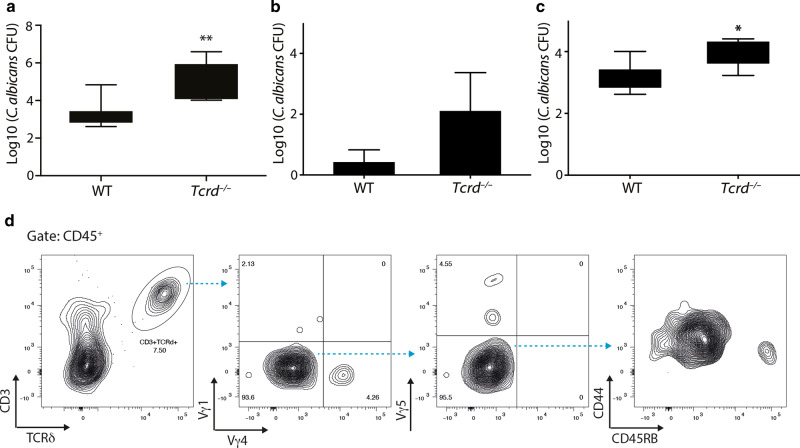
C57BL/6J and *Tcrd*^−/−^ females (*n* = 5–8) were infected intravaginally with *C. albicans* 529 L and fungal burden assessed 7 days post infection in vaginal lavage and uterine lysate samples. **a** Combined vaginal and uterine fungal burden. **b** Uterine fungal burden. **c** Vaginal fungal burden. **d** TCR usage and surface immunophenotype of vaginal γδ T cells was determined by flow cytometric analysis of Vγ1, Vγ4 and Vγ5 chains and CD44 and CD45RB. Graphs indicate mean ± SD.

First, Dr. Fidel points out that VVC is normally restricted to the vaginal space. Indeed, this is what we observed in most wild type mice; however, following *Candida* challenge of *Tcrd*^−*/*−^ animals, we observed increased fungal colonisation of the uterus (Fig. 1a, b), suggestive of ascending infection. Thus, a strong possibility exists that γδ T cells, conceivably those in the uterus, naturally limit VVC. Additionally, this observation formed the basis for our decision to report the combined uterine and vaginal burden (Fig. 7 of ref. ^1^). Nonetheless, when this is teased out, γδ T cell-deficient mice show increased burdens in both sites (Fig. 1a–c). Note that measurement of the vaginal fungal burden, was performed by vaginal lavage with 100 μL of sterile PBS, of which serial dilutions were plated in triplicate into YPD chloramphenicol plates, as is common practice.^5^

Consistent with earlier reports,^2^ when characterising immune infiltration into the vaginal compartment, we noted that a substantial fraction of leucocytes comprised γδ T cells which mostly shared their TCR usage (Vγ1^−^4^−^5^−^) and surface immunophenotype with uterine γδ T cells (Fig. 1d). Thus, future studies may usefully determine the relative contributions of vaginal and uterine γδ T cells to limiting *Candida* infection in the FRT and whether the γδ T cells at the two sites dynamically intermix.

Second, as Dr. Fidel points out, there is still need to elucidate the precise contributions of defined effector molecules to the control both of *Candida* infection and of consequential disease. We noted in our paper that a link to IL-17, produced by FRT γδ T cells, was attractive because IL-17 is a profound neutrophil regulator in mice, because *Candida-*infected *Tcrd*^*−/*−^ mice showed significantly fewer neutrophils in the vaginal cell suspensions; and because T cell production of IL-17 was previously reported to be protective in candidiasis^6^ in which it was associated with neutrophil infiltration and the production of antimicrobial factors. However, this clearly merits re-evaluation in light of the study recently published by Peters et al. showing that IL-17RA, Act1 and IL-22 deficiency did not impact fungal burden or tissue pathology in their VVC model.^7^ This notwithstanding, we clearly acknowledged in our paper that the effector mechanisms by which γδ T cells regulate candidiasis were unknown and specifically suggest in the Discussion section, that other possibilities, such as the promotion of tissue repair, exist.

Finally, with regards to the discrepancy between our findings and those published by Dr. Fidel’s group on the role of γδ T cells in protection to VVC,^8^ we can only suggest that variations in animal housing, microbiome composition, *C. albicans* strain and/or inoculation may collectively or individually contribute to the differences observed. Unfortunately, there is precedent for such discrepancies in animal models of inflammation in which γδ T cells are implicated, as illustrated by studies of imiquimod-induced dermatitis and their discussions.^9–11^

In sum, the findings presented in our published study reflect carefully conducted experiments, data from which clearly support the conclusions drawn. In several respects, those conclusions have provided important new insights into murine uterine γδ T cells. While it is not necessarily the case that such cells will, in all settings, provide non-redundant protection in response to *Candida* infection, there is a strong likelihood that their effector molecules will include those promoting barrier integrity that may increase resistance to FRT infections and to environmental challenges more generally. In this regard, the uterus shares similarities with the skin and gut that have been much more fully explored.
